# Spatial Variation in Abundance, Size and Orientation of Juvenile Corals Related to the Biomass of Parrotfishes on the Great Barrier Reef, Australia

**DOI:** 10.1371/journal.pone.0057788

**Published:** 2013-02-28

**Authors:** Melanie L. Trapon, Morgan S. Pratchett, Andrew S. Hoey

**Affiliations:** ARC Centre of Excellence for Coral Reef Studies, James Cook University, Townsville, Queensland, Australia; University of California San Diego, United States of America

## Abstract

For species with complex life histories such as scleractinian corals, processes occurring early in life can greatly influence the number of individuals entering the adult population. A plethora of studies have examined settlement patterns of coral larvae, mostly on artificial substrata, and the composition of adult corals across multiple spatial and temporal scales. However, relatively few studies have examined the spatial distribution of small (≤50 mm diameter) sexually immature corals on natural reef substrata. We, therefore, quantified the variation in the abundance, composition and size of juvenile corals (≤50 mm diameter) among 27 sites, nine reefs, and three latitudes spanning over 1000 km on Australia’s Great Barrier Reef. Overall, 2801 juveniles were recorded with a mean density of 6.9 (±0.3 SE) ind.m^−2^, with *Acropora, Pocillopora*, and *Porites* accounting for 84.1% of all juvenile corals surveyed. Size-class structure, orientation on the substrate and taxonomic composition of juvenile corals varied significantly among latitudinal sectors. The abundance of juvenile corals varied both within (6–13 ind.m^−2^) and among reefs (2.8–11.1 ind.m^−2^) but was fairly similar among latitudes (6.1–8.2 ind.m^−2^), despite marked latitudinal variation in larval supply and settlement rates previously found at this scale. Furthermore, the density of juvenile corals was negatively correlated with the biomass of scraping and excavating parrotfishes across all sites, revealing a potentially important role of parrotfishes in determining distribution patterns of juvenile corals on the Great Barrier Reef. While numerous studies have advocated the importance of parrotfishes for clearing space on the substrate to facilitate coral settlement, our results suggest that at high biomass they may have a detrimental effect on juvenile coral assemblages. There is, however, a clear need to directly quantify rates of mortality and growth of juvenile corals to understand the relative importance of these mechanisms in shaping juvenile, and consequently adult, coral assemblages.

## Introduction

Most marine organisms have open populations, where rates of settlement are decoupled from local abundance and fecundity of adult individuals [1], [2]. Replenishment and persistence of marine populations is therefore, dependent upon the supply of pelagic larvae, their successful settlement into reef habitats, and the subsequent growth and survival of individuals until they reach sexual maturity and enter the adult population (e.g., marine invertebrates: [1], [3], [4]; corals: [5], [6]; fish: [7]–[10]). A plethora of studies have examined settlement patterns of scleractinian corals, mostly using artificial substrates, and showed that settlement rates are highly variable, both in space and time [11]–[14]. These patterns established at settlement may however, be modified substantially by post-settlement processes such as differential growth and survivorship [15]–[17]. Consequently, spatial patterns in coral settlement often bear little resemblance to patterns of adult coral abundance [13], [18]. Most notably, Hughes et al. [13] found that settlement rates of scleractinian corals varied by an order of magnitude along 2,000 km’s of Australia’s Great Barrier Reef, yet adult coral cover was very consistent among the five latitudinal regions studied. Hughes et al [13] suggested that these findings were due to large-scale variations in early post-settlement dynamics, which compensate for marked differences in settlement rates. This apparent disconnect between larval settlement and adult coral populations is poorly understood, and only few studies have focused on early life-stages of corals on natural substrata, mainly due to difficulties associated with identifying small colonies on natural substrates [19].

Corals are typically very small at settlement (≤2 mm, [20]), and very difficult to detect *in situ*. Mortality of these corals is also recorded to be very high immediately following settlement, often reaching 99% within the first months post-settlement [6], [21], [22], which will have a marked influence on the distribution of later stages of juvenile corals, operationally defined as visible colonies from 10 to 50 mm diameter [23]–[25]. Based on size at settlement and current estimates of coral growth, the age of these juvenile corals would range from 2 to 7 years depending on the taxa [20], [26]–[28]. Juvenile corals are also subject to high mortality [21], [24], [29], but as mortality rates often decrease with increasing size of coral colony [30], the distribution of juvenile corals may be a better predictor of the distribution, abundance and composition of coral populations.

High incidences of juvenile coral mortality are often attributed to predation or incidental grazing by fishes [24], [31]–[33], and/or overgrowth or smothering by macroalgae [34]. This results in a potentially complex, and probably non-linear, relationship between juvenile survivorship of scleractinian corals and local abundance of herbivorous fishes; moderate levels of herbivory can have beneficial effects on coral survivorship in term of reducing algal cover and opening new space for coral to settle, thus maintaining coral dominated reefs [34]. However, high densities and intensive feeding activity by grazing parrotfishes may actually lead to increased levels of incidental mortality for juvenile corals [24]. Settlement into cryptic habitats has been suggested to be a key strategy by juvenile corals to reduce predation and susceptibility to grazing [35], thereby increasing post-settlement survival [36], [37]. However, corals that settle within cryptic micro-habitats may be sheltered from sunlight and experience reduced growth. It is possible therefore, that micro-habitat preferences of juvenile corals also vary with respect to the risk of predation, due to variation in the local abundance of grazing parrotfishes.

The purpose of this study was to quantify the spatial variation in abundance of juvenile corals (≤50 mm) among three sectors of the Australian’s Great Barrier Reef that differ in their latitude (14° S, 18° S and 23° S) and to compare these patterns to spatial variation in abundance of parrotfishes. Scraping and excavating parrotfishes (f. Labridae), unlike roving herbivorous fishes from the Acanthuridae, Siganidae and Kyphosidae, remove parts of the underlying substratum when feeding. Consequently, incidental grazing by scraping and excavating parrotfishes may be an important source of mortality for recently settled and juvenile corals. Specifically, the abundance, composition, size, and orientation of juvenile coral assemblages were compared among sites (within reefs), among reefs, and among latitudinal locations, along the Australian’s Great Barrier Reef. Little is known about the juvenile life-stage of corals on natural substrata, thus this study provides important ecological data on early life-history of scleractinian corals at small and large scales.

## Methods

### Ethics Statement

The activities for this study were conducted under permission from the Great Barrier Reef Marine Park Authority (Permit Number G09/32834.1). Only visual censuses of fish and benthic communities were conducted during this study; no fauna or flora were collected or manipulated.

### Study Sites

**Figure 1:**
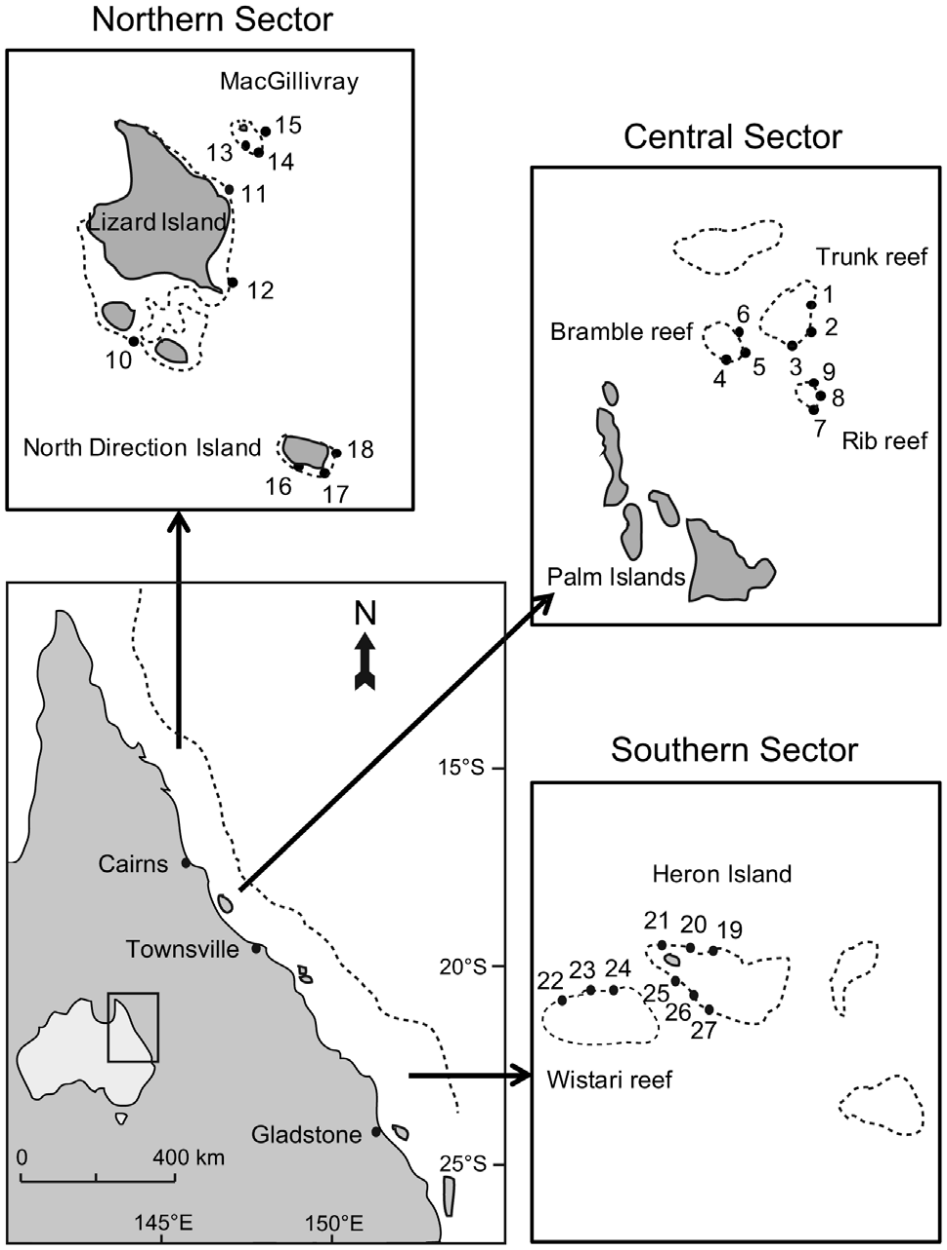
Map of Eastern Australia showing the Great Barrier Reef region with the three sectors chosen for this study. At each sector, three mid-shelf reefs with three sites each on the reef crest have been surveyed.

Surveys of juvenile corals were conducted in three distinct locations on the Great Barrier Reef (GBR) from north to south, separated by at least 500 km: i) northern GBR, in the vicinity of Lizard Island (14°41′S, 145°28′E), central GBR, in vicinity of Trunk Reef (18°25′S, 146°47′E), and southern GBR, in the vicinity of Heron Island (23°27′S, 155°55′E) (Fig. 1). Within each location, sampling was conducted at three reefs, and three sites per reef, giving a total of nine sites per location. Only mid-shelf reefs were sampled to minimize any effects of cross-shelf variation, and all sampling was constrained to a single habitat type, the exposed reef crest. The exposed reef crest was selected as this habitat is characterised by hard substratum covered by i) short sparse turf algae with a conglomeration of detritus, microbes, small invertebrates and microalgae, also referred as “epilithic algal matrix” (EAM, see [38]), with underlying CCA (crustose coralline algae), making the distinction between turf algae and CCA very difficult, ii) high cover of adult corals [39] and iii) high rates of coral recruitment [14]. The biotic and abiotic processes that may influence the distribution of juvenile corals operate across a range of spatial scales [13]. Therefore, this hierarchical nested sampling design facilitates the examination of local and regional variation in juvenile coral assemblages, and provides greater insight into the processes that may be structuring these populations on the GBR.

### Juvenile Corals Census

At each site, three replicate 10-m transects were established on the crest, parallel with depth contours and separated by 1 to 10 m. Five 1 m^2^ quadrats were placed randomly along each transect, giving a total of 405 quadrats. Juvenile scleractinian corals were defined as any colonies visible with the naked eye with a maximum diameter of 50 mm, following Rylaarsdam [25] and showing distinct growth and morphological characteristic (e.g., base approximately round). Care was taken to exclude colonies resulting from fission, shrinkage or fragmentation of older colonies [40]. To maximize detection of juvenile corals, the 1-m^2^ quadrats were divided into a 10×10 grid using strings placed at 10 cm intervals along the vertical and horizontal axes. The resulting one hundred 10 cm^2^ squares were systematically inspected for the presence of juvenile corals. All juvenile corals detected were identified to the highest possible taxonomic level (mostly genus) and the maximum diameter measured to the nearest millimetre using calipers. The smallest corals detected were 5 mm diameter, but only a very small proportion (2.4%) of juvenile corals were <10 mm, reflecting difficulties in detecting very small corals with the naked eye. All juveniles were also examined for any signs of damage, however, the level of partial mortality was extremely low across sectors (relative proportion: 1.4%, 2.7% and 1.8% within the northern, central and southern sectors respectively) and therefore no further consideration was undertaken.

To determine if juvenile corals were associated with specific microhabitats, the orientation of each juvenile coral was recorded. The orientation was classified into one of four categories: (i) horizontal - the substratum on which the juvenile was attached had an angle <45°; (ii) vertical - the substrate had an angle >45°; (iii) immersed - the juvenile was positioned below the level of the surrounding substrate, either inside a crevice or among the branches of a recently dead coral; (iv) covered - the juvenile had settled beneath an existing structure (e.g., a tabulate coral).

### Adult Coral Census

To determine if coral cover influenced the density of juvenile corals, adult cover and composition was recorded within the same quadrats used to quantify juvenile coral assemblages. A total of 81 regularly spaced points formed by the 10×10 grid were surveyed within each quadrat. Any scleractinian (hard) corals underlying each survey point were identified to genus. Other benthic components such as soft corals (1.9±0.35 SE %), macroalgae (0.3±0.05 SE %) and sand/rubble (1.6±0.21 SE %) cover were extremely low on the reef crest, characteristic of this habitat, thus they were not included in the data analysis.

### Parrotfish Census

Species-level surveys of parrotfishes were conducted using underwater visual censuses along a series of 50-m belt transects at each site. Each transect consisted of a diver swimming along the reef crest and recording all parrotfishes greater than 10 cm total length (TL) within a 5 m wide belt while simultaneously deploying a 50 m transect tape. This procedure minimised disturbance prior to censusing and allowed a specified area to be surveyed. Individual fishes were identified to species and placed into 5cm size categories. Care was taken not to re-census fish that left and subsequently re-entered the transect area. Eight transects were surveyed within each site on each reef (total n = 216 transects). Fish densities were converted to biomass using published length-weight relationships for each species, following Hoey and Bellwood [41].

Parrotfishes may be categorised into two groups based on the amount of substratum that is removed through the feeding action: 1) scrapers and excavators; 2) macroalgal browsers [42]. Scraping and excavating parrotfishes (i.e., *Cetoscarus bicolor, Chlorurus* spp., *Hipposcarus longiceps, and Scarus* spp.) remove pieces of the carbonate substratum when feeding and subsequently may incidentally remove or damage recently settled or small juvenile corals. In contrast, the macroalgal browsing parrotfishes (i.e., *Calotomus* spp. and *Leptoscarus vaigiensis*) remove only algae and associated detrital material and are unlikely to cause any direct mortality of juvenile corals. Browsing parrotfishes are rare on the GBR [42], and none were recorded during the visual surveys within each of the three regions. Consequently, our analyses were restricted to scraping and excavating parrotfishes.

### Statistical Analysis

Spatial variation in the abundance of juvenile corals, cover of adult corals and herbivorous fish biomass were examined using hierarchically nested analysis of variance (ANOVA), with sites nested within reef and reefs nested within latitudinal sectors. Juvenile coral abundance and fish biomass were log_10_ (x+1) transformed and adult coral cover was arcsine-square root transformed to improve the homoscedasticity and normality. To examine spatial variations in the assemblage structure of juvenile and adult corals a hierarchically nested multivariate analysis of variance (MANOVA) was used. The analyses were based on the abundance and cover of the three dominant genera (i.e, *Acropora*, *Pocillopora*, *Porites*) and ‘other’ scleractinian corals.

Bivariate correlations were used to test for any relationship between the abundance of juvenile corals (≤50 mm) and the cover of scleractinian coral, and the biomass of scraping and excavating parrotfishes. Furthermore, correlations were used to test for an effect of parrotfish biomass on the density of juvenile corals occurring on horizontal, immersed, under and vertical surfaces.

Chi-squared tests were used to determine whether orientation (i.e., horizontal, vertical, immersed, and under) and size structure of juvenile coral assemblages differed among latitudinal sectors (i.e., northern, central, and southern GBR). For the size structure, juvenile corals were placed into 5 mm size classes; ≤14, 15–19, 20–24, 25–29, 30–34, 35–39, 40–44, 45–50 mm.

## Results

### Juvenile Corals

**Figure 2:**
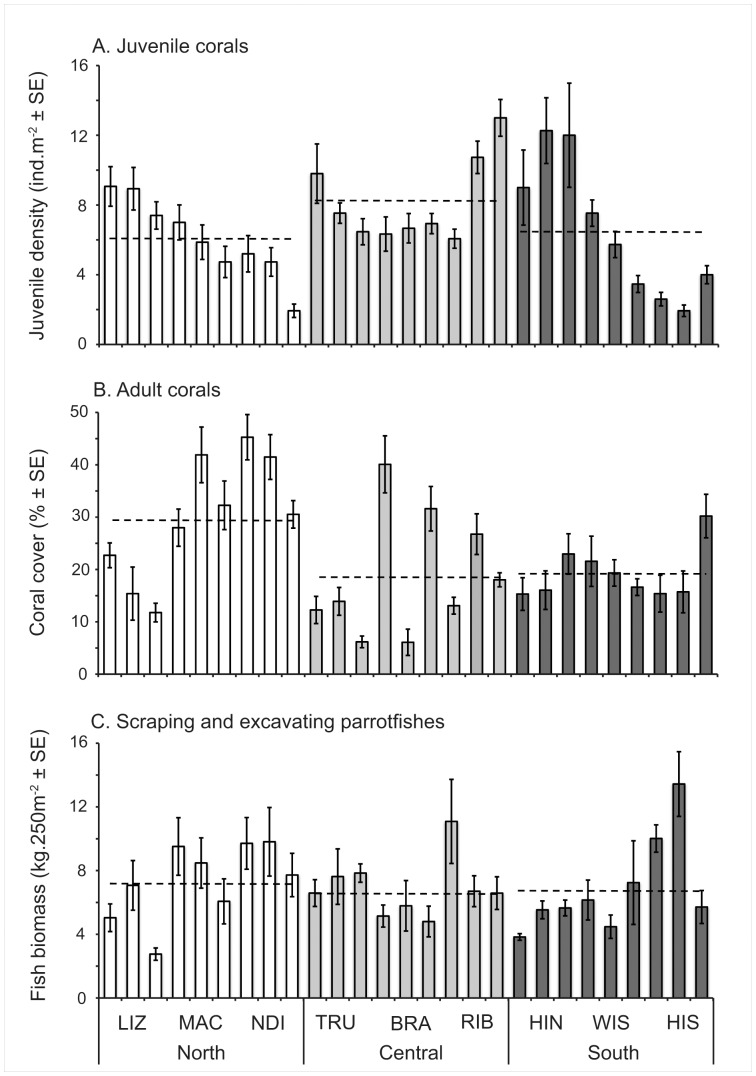
Mean (A) juvenile densities, (B) coral cover, and (C) biomass of scraping and excavating parrotfishes at Lizard Island (LIZ), Macgillivray (MAC) and North Direction Island (NDI) reefs (northern sector, *white*), Bramble (BRA), Rib (RIB), and Trunk (TRU) reefs (central sector, *light grey*) and Heron Island Nord (HIN), Wistari (WIS) and Heron Island South (HIS) reefs (southern sector, *dark grey*), for three different sites at each reef. The error bars represent 95% confidence intervals. Dashed line represents the overall mean for each sector.

**Figure 3:**
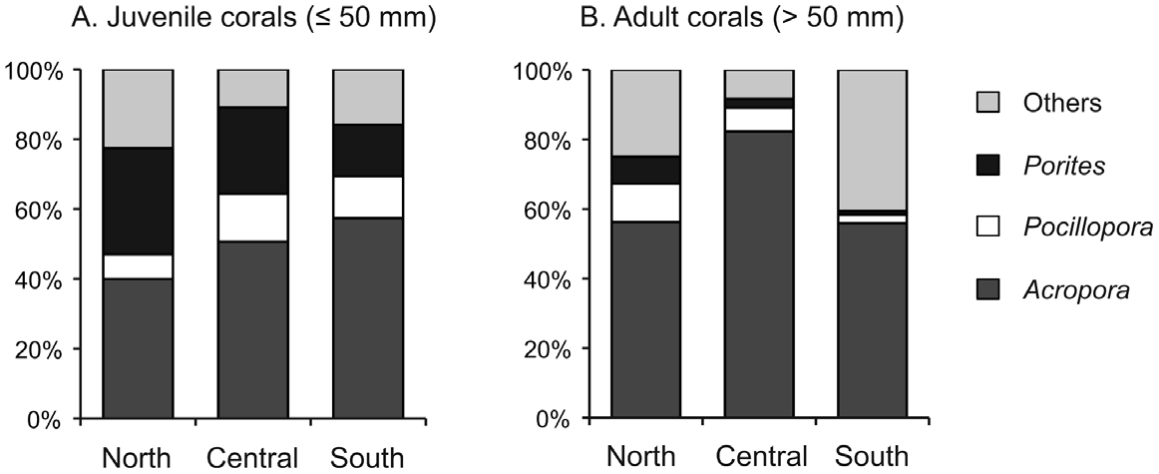
Relative abundance of *Acropora*, *Pocillopora*, *Porites* and other corals in (A) the juvenile (≤50 mm) and (B) adult assemblages among the three sectors.

A total of 2,801 juvenile corals, from 28 genera and 8 families, were recorded across all sites, giving a mean of 6.92 juveniles ±0.25 SE per m^2^. Densities of juvenile corals ranged from 0 to 38 per m^2^ among quadrats, and was extremely variable even among quadrats situated along the same transect. The densities of juvenile corals varied significantly among reefs and sites, but displayed no significant variation among latitudes. Most of the variation (62.6%) occured within sites (Table 1A). Variation among reefs was most pronounced in the southern GBR where mean juvenile density varied 3.9-fold, from 2.8±0.3 ind.m^−2^ on Heron Island South to 11.08±1.4 ind.m^−2^ on Heron Island North (Fig. 2A). Juvenile assemblages were dominated by three genera (*Acropora*, *Pocillopora*, and *Porites*) that collectively accounted for 84.1% of all juveniles recorded. Taxonomic composition varied significantly among sectors, reefs and sites (Table 2A), with relative proportions of juvenile *Acropora* higher at the southern sector (57.4%), *Pocillopora* corals higher at the central sector (13.7%) and *Porites* corals higher at the northern sector (30.5%, Fig. 3A).

**Table 1:** Results of hierarchically-nested ANOVAs examining variation in (A) density of juvenile corals, (B) adult coral cover, and (C) biomass of scraping and excavating parrotfishes, among latitudes, reefs, and sites.

A. Juvenile corals						
Source	SS	df	MS	F	p	Var (%)
Sector	2.340	2	1.170	0.972	0.431	0.0
Reef (Sector)	7.221	6	1.203	6.277	0.001	26.4
Site (Sector*Reef)	3.451	18	0.192	3.618	0.000	11.0
Residual	20.029	378	0.053			62.6
**B. Adult corals**						
**Source**	**SS**	**df**	**MS**	**F**	**p**	**Var**
Sector	1.962	2	0.981	2.318	0.180	8.4
Reef (Sector)	2.539	6	0.423	2.163	0.096	10.4
Site (Sector*Reef)	3.522	18	0.196	6.855	0.000	22.8
Residual	10.791	378	0.029			58.4
**C. Parrotfishes biomass**						
**Source**	**SS**	**df**	**MS**	**F**	**p**	**Var**
Sector	0.013	2	0.007	0.015	0.985	0.0
Reef (Sector)	2.652	6	0.442	4.234	0.008	12.7
Site (Sector*Reef)	1.879	18	0.104	1.744	0.035	7.4
Residual	11.312	189	0.060			79.9

Juvenile coral densities and fish biomass were Log_10_ (x+1) transformed and coral cover data were arcsine-square root transformed.

**Table 2:** Multivariate analyses of variance to test for variation in taxonomic composition of (A) juvenile corals, and (B) adult corals among latitudes, reefs, and sites.

A. Juvenile corals					
Effect	Pillai’s Trace	F	df	Error df	p
Sector	0,264	14,314	8	752	0.000
Reef (Sector)	0,624	11,653	24	1512	0.000
Site (Sector*Reef)	0,452	2,672	72	1512	0.000
**B. Adult corals**					
**Source**	**Pillai’s Trace**	**F**	**df**	**Error df**	**p**
Sector	0,732	54,236	8	752	0.000
Reef (Sector)	0,473	8,452	24	1512	0.000
Site (Sector*Reef)	0,567	3,470	72	1512	0.000

Juvenile coral abundances were log_10_ (x+1) transformed and coral cover was arcsine-square root transformed.

**Figure 4:**
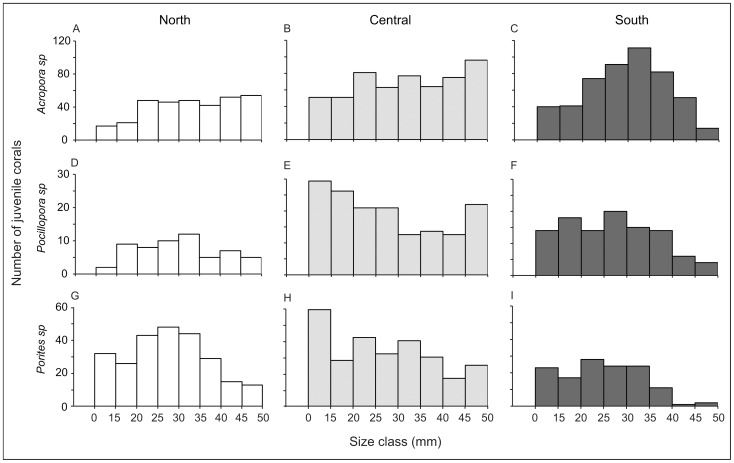
Size-class frequency distribution (mm) of juvenile corals ≤50 mm from the three main taxa: (A, B, and C) *Acropora*, (D, E and F) *Pocillopora,* and (G, H and I) *Porites sp*, at the northern (*white*), central (*light grey* ) and southern (*dark grey*) sectors of the GBR. Juveniles <10 mm have been added to the size class 10–14 mm.

The size structure of juvenile *Acropora*, *Pocillopora*, and *Porites* corals differed significantly among latitudinal sectors (Chi-square contingency Table 3A, Fig. 4). Juvenile *Acropora* were relatively evenly distributed among size classes in the northern and central sectors (Fig. 4a, b), whereas in southern sector the highest frequency of individuals was in the size-class 30–34 mm (relative proportion: 22%) with few individuals in the smallest (7.9%) and largest (2.8%) size classes (Fig. 4c). The size distribution of juvenile *Pocillopora* and *Porites* displayed some similarities among sectors. In the northern sector juvenile *Pocillopora* and *Porites* peaked in the 25–29 and 30–34 mm size classes (25–29 mm: 17.2% and 19.2% respectively; 30–34 mm: 20.7% and 17.6% respectively), and the relative proportion of individuals decreased with size (Fig. 4d, g), while in the central sector frequencies were highest in the smallest size class (18.5% and 21.6%, respectively) and generally decreased with size (Fig. 4e, h). In the southern sector juvenile *Pocillopora* and *Porites* were relatively evenly distributed among size classes up to 40 mm, with few individuals in the two largest size classes (3.8% and 1.5% respectively; Fig. 4f, i).

**Table 3:** Chi-square tests examining latitudinal variation in (A) size-class frequencies and (B) surface orientation of juvenile corals from the three main genera.

A. Size-Class			
Genera	χ^2^	df	p
*Acropora*	91.20	14	0.000
*Pocillopora*	24.34	14	0.041
*Porites*	30.39	14	0.006
**B. Surface orientation**			
**Genera**	**χ^2^**	**df**	**p**
*Acropora*	67.66	6	0.000
*Pocillopora*	42.78	6	0.000
*Porites*	21.46	6	0.002

Juveniles <10 mm have been added to the size class 10–14 mm due to the difficulty to observe such small corals on natural substrate. The size-classes are as follow: ≤14, 15–19, 20–24, 25–29, 30–34, 35–39, 40–44, 45–50 mm, and the orientation on the natural substrate are: horizontal, immersed, under and vertical.

**Figure 5:**
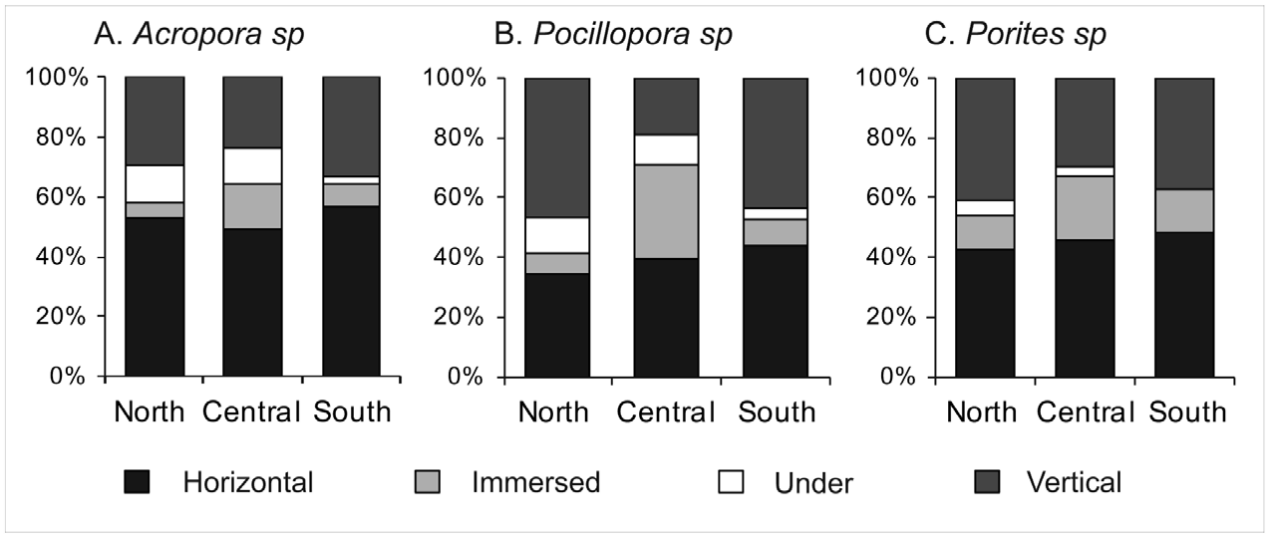
Comparison of surface orientation use by juvenile scleractinian corals across the northern (n = 135), central (n = 135) and southern reefs (n = 135) on the GBR, for (A) *Acropora sp,* (B) *Pocillopora sp*, and (C) *Porites sp*.

The majority of juvenile corals surveyed in all sites, reefs and sectors were recorded on horizontal (47.5%) and vertical (32.5%) surfaces, but orientation of the three main genera varied among sectors (Table 3B; Fig. 5). In the central GBR, juvenile *Acropora, Pocillopora* and *Porites* were found less often on vertical surfaces (23.8%, 19.2%, and 29.5%, respectively) and more often immersed in crevices (14.9, 31.1, and 21.5%, respectively) compare to the northern or southern reefs (Fig. 5). In contrast, the occurrence of juvenile *Acropora, Pocillopora* and *Porites* under existing structures was low especially on the southern reefs (2.8%, 3.8%, and 0%, respectively), compared to the central (11.8%, 9.9%, and 3.3%) and northern (12.5%, 12.1%, and 5.2%) reefs (Fig. 5).

### Adult Corals

Mean adult coral cover ranged from 29.9±1.5% in the northern location to 19.2±1.2% and 18.7±1.4% in the southern and central sectors respectively. However, large variation in coral cover within (58.4%) and among sites (22.8%) precluded the detection of any significant variation among latitudinal sectors or reefs (Table 1B; Fig. 2B). *Acropora*, *Pocillopora* and *Porites* dominated the adult coral assemblages, collectively accounting for more than 80% of total coral cover. Taxonomic composition of adult corals varied significantly among latitudinal sectors, reefs and sites (Table 2B), with relative abundance of *Acropora* corals higher at the central sector (82.3%) than in the northern (56.2%) and southern (55.9%) sectors (Fig. 3B). Conversely, the relative abundance *Porites* and *Pocillopora* was higher in the central and northern sectors, compared to the central sector (Fig. 3B).

### Parrotfish Communities

Overall, the mean biomass of scraping and excavating parrotfishes was 7.1±0.3 kg.250 m^−2^ (Fig. 2C). Despite significant variation in the biomass of parrotfishes among reefs and sites with most of the variation within sites (79.9%), there was no variation among sectors, ranging from 6.89±0.53 kg.250 m^−2^ in the southern sector to 6.90±0.48 and 7.35±0.54 kg.250 m^−2^ in the northern and central sectors respectively (Table 1C).

### Relationship among Variables

**Figure 6:**
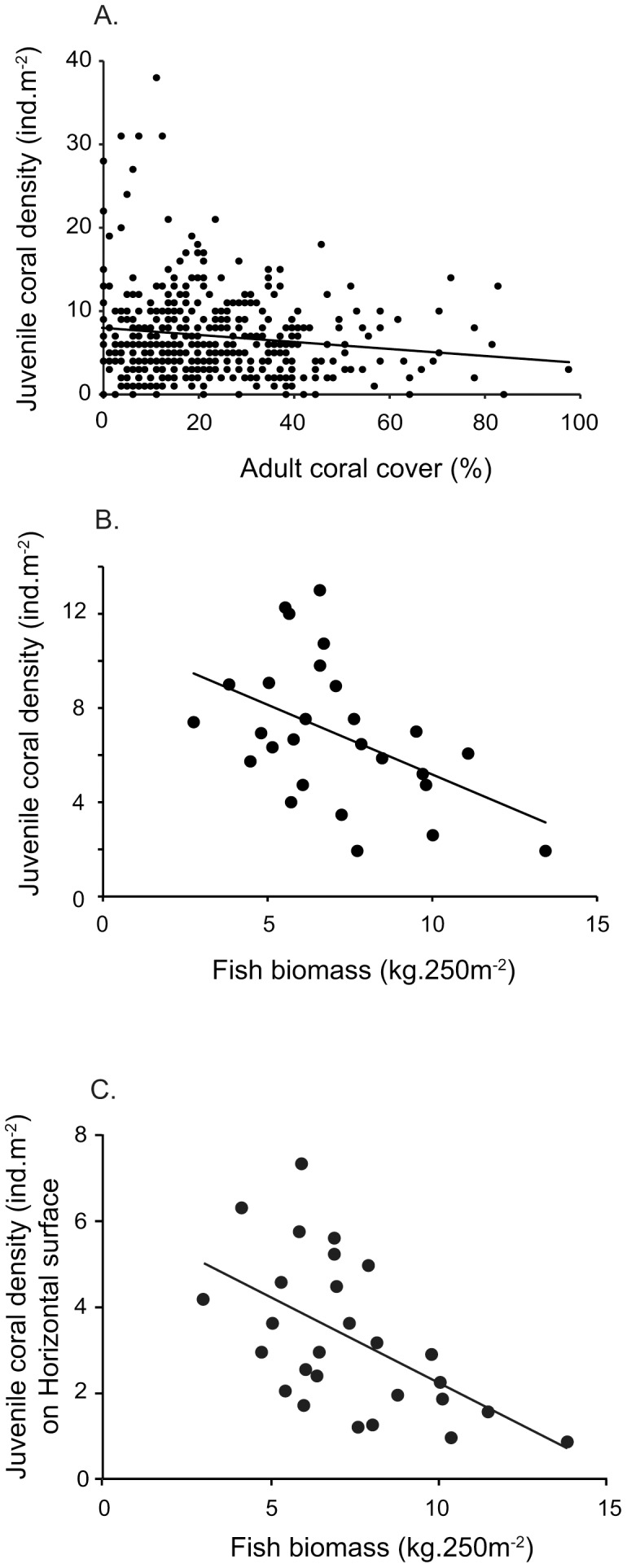
Relationship between (A) juvenile coral density and adult coral cover at quadrat scale, (B) juvenile coral density and the biomass of scraping and excavating parrotfishes at site scale, and (C) juvenile coral density on horizontal surfaces and the biomass of scraping and excavating parrotfishes at site scale.

Density of juvenile corals was weakly negatively correlated to coral cover at the scale of quadrat only (r = −0.128, N = 405, p = 0.01, Fig. 6A), but adult coral cover explained only 1.6% of the variation in juvenile densities. Parrotfish biomass explained 21.7% of the variation in total juvenile density (r = −0.466, N = 27, p = 0.014; Fig. 6B) but this was even higher (34.7%) when considering only juvenile corals occurring on horizontal surfaces (r = −0.589, N = 27, p = 0.001; Fig. 6C). In contrast, there was no significant relationship between parrotfish biomass and the density of juvenile corals on immersed (r = −0.230, N = 27, p = 0.249), under (r = 0.090, N = 27, p = 0.656) and vertical (r = −0.311, N = 27, p = 0.115) substrates.

## Discussion

This study revealed significant fine-scale spatial heterogeneity in the density, taxonomic composition, size-class distribution and orientation of juvenile corals among reefs of the GBR. The density of juvenile corals was highly variable both among reefs and sites, with most of the variation occurring within site, but displayed limited variation over the larger latitudinal scale. Fine-scale variation in the abundance of juvenile corals may be influenced by local scale hydrodynamic regimes [43], disturbance history [11], larval supply [13], habitat availability [44] and predation [24]. Overall abundance of juvenile corals was strongly and negatively correlated with the biomass of scraping and excavating parrotfishes, especially when considering only juvenile corals occurring on horizontal surfaces. This suggests that patterns of post-settlement mortality exert a strong influence on patterns of juvenile abundance, either augmenting or obscuring patterns established at settlement. The relative importance of larval supply versus post-settlement mortality is likely to vary temporally and spatially and would need to be explicitly tested using manipulative experiments.

Previous studies [5], [13] have suggested that there are strong latitudinal differences in the underlying dynamics of scleractinian corals on the GBR based on marked geographical differences in settlement rates despite similar levels of adult abundance. Both large-scale sampling and a meta-analysis of small-scale studies have found significant latitudinal variation in rates of coral settlement to artificial substrata [13], [45]. Most notably, settlement rates in the region of Lizard were an order of magnitude higher compared to reefs in the region of Heron Island [13], [45]. These apparent differences between settlement rates and juvenile abundance are attributable to inherent (e.g., taxonomic) biases in coral settlement to artificial substrates, which is further confounded by failing to take account of early post-settlement mortality [24]. If latitudinal differences in settlement rates [13] reflect large-scale variation in larval supply [45] then these differences must be offset by increased survivorship of juvenile corals with increasing latitude.

While the overall abundance of juvenile corals was very consistent among latitudinal locations, the size structure varied significantly among latitudes for *Acropora*, *Pocillopora*, and *Porites*. *Acropora* juveniles were distributed relatively evenly among size-classes in the northern and central sectors, with higher than expected abundance of larger individuals (≥40 mm) given expected attrition through increasing size-classes. In contrast, relative abundances of *Pocillopora* and *Porites* juveniles were distributed evenly in the northern and southern sectors with abundance decreasing toward the larger size classes, whereas in the central sector, *Pocillopora* and *Porites* juveniles were more abundant within the smallest size-class (<15 mm), and still well represented in the largest size-class (45–50 mm). Large juveniles (45–50 mm) were far less abundant for all three genera in the southern sector, suggesting higher post-settlement mortality and/or slower growth compared to juvenile corals in the northern and central sectors. If so, post-settlement growth and mortality would be expected to augment, not offset, the latitudinal variation in settlement rates, but direct measures of growth and mortality (especially among the smallest size-classes) are needed to assess large-scale variation in demographic rates from juvenile corals. However, the difficulty in detecting small corals, especially <15 mm, significantly limits the capacity to measure early post-settlement mortality *in situ*. In this study, despite thoroughly searching for all juveniles under 50 mm diameter, we certainly under-estimated the local abundances of individuals in the smallest size classes (<15 mm), but this bias was assumed to be constant and should not affect overall conclusions.

Juvenile corals were found more often on horizontal surfaces, but the proportion found on vertical, under a coral or immersed surface changed greatly between sectors. This could suggest that coral larvae select different suitable orientation surfaces depending on biotic and abiotic conditions of the local environment and habitat complexity they encounter. Studies on larval settlement choice and ultimately juvenile corals orientation on natural and artificial substrates have shown that in shallow water, coral larvae preferentially settle on vertical and under surfaces as opposed to upward horizontal substrates [14], [37], [46], [47], probably to avoid sedimentation, incidental grazing and overgrowth by algae which are known to limit recruit survival and growth [35], [48]. However, these studies also found that once the juvenile colony reaches a certain size, growth and survival may be maximised on horizontal surfaces (e.g. escape in size: [21]). This suggests that it may be beneficial for coral larvae to settle in cryptic micro-habitats such as crevices, and then outgrow the micro-habitat to become orientated horizontally on the substrate [49]. Although more than half of the juveniles observed in this study occurred on horizontal surface, the availability of the four different orientations was not recorded, which could have further reinforced the data. We therefore, cannot predict whether differences in size structure across sectors are function of substrate orientation, or whether larvae preferably settled on a certain orientation surface. However, we can infer that horizontal surfaces might offer a better chance for survival once the juvenile coral grow in the open, based on the literature cited above.

Similar to juvenile density and adult coral abundance, the biomass of scraping and excavating parrotfishes did not vary among latitudes but displayed considerable variation among and within reefs. A striking result was the negative correlation between the biomass of parrotfish and the density of juvenile corals across all sites. Explicitly, sites with high parrotfish biomass tended to have low juvenile densities, and less juveniles occurring on horizontal surfaces. Although biomass does not equate to the functional impact (i.e., area grazed) of individual parrotfishes per se, it does provide a useful proxy in the absence of species- and size-specific feeding rates and bite sizes. While larger parrotfishes have been shown to scrape a disproportionately larger area of reef substratum per bite than smaller individuals [50], [51], the volume of material removed per unit body mass is relatively consistent for parrotfish greater than 10 cm TL [51]. Incidental grazing by parrotfishes has been found to reduce the survival of corals within the first few weeks after settlement [24], [52], but not larger more established juvenile corals [24]. Therefore, parrotfishes might have indirectly influenced juvenile densities observed in this study by incidentally grazed on earlier smaller cohorts, decreasing their survival, which in turn resulted in a negative relationship between juvenile densities and parrotfish biomass.

It is widely accepted that scraping and excavating parrotfish are a key functional group on coral reefs, mediating the competition for space between corals and algae and maintaining healthy reef systems by clearing space on the substrate for new coral recruits [34], [53]. While the positive effects of these herbivorous fishes on reef processes are well established, the potential deleterious effects are poorly understood. The vast majority of parrotfish feed almost exclusively on crustose coralline algae, algal turfs and associated detritus [41], [54], also called epilithic algal matrix (EAM, see 38). However, through their feeding actions parrotfish may also incidentally consume and/or damage small juvenile corals. At low biomass, scraping and excavating parrotfishes have been found to enhance coral settlement on a subtropical reef [55], however on the Great Barrier Reef, parrotfishes are far more abundant and may account for over 80% of the total biomass of herbivorous fish in some habitats [56], [57]. It appears likely that at very high biomass, any positive effects of clearing space on the substrate are negated by high levels of incidental predation. This was further supported by a negative correlation between parrotfish biomass and the number of juvenile corals occurring on horizontal surface, which are the most susceptible to grazing. Incidental predation of juvenile corals by parrotfish, along with many other important factors not tested in this study (e.g. abundance and type of CCA; [58]), may be ecologically important in structuring juvenile coral assemblages on the Great Barrier Reef.

This is the first large-scale study of coral recruitment, testing for large (latitudinal) and small (site) level differences in the abundance of juvenile corals on natural substrates, thereby complementing previous studies that looked at hierarchical patterns of coral settlement. Despite marked latitudinal variation in larval supply and settlement rates reported previously [13], [45], we found no large-scale differences in abundance of juvenile corals. This suggests that latitudinal variation in coral settlement may be highly modified by post-settlement processes, whereby low settlement rates in the southern sectors could be offset by high post-settlement survival. The size frequency distribution of juvenile corals actually suggests that there is lower (not higher) post-settlement survivorship and/or slower growth in the southern sector. However, direct measure of mortality and growth rates of juvenile corals at this hierarchy of spatial scales is critical if we are to understand latitudinal variation in the population dynamics of coral population and the factors influencing replenishment and resilience.
